# Prediction of rapid maxillary expansion by assessing the maturation of the midpalatal suture on cone beam CT

**DOI:** 10.1590/2177-6709.21.6.115-125.sar

**Published:** 2016

**Authors:** Fernanda Angelieri, Lorenzo Franchi, Lucia H. S. Cevidanes, Bruno Bueno-Silva, James A. McNamara

**Affiliations:** 1 Assistant Professor, Guarulhos University, Guarulhos, Brazil; and Visiting Scholar, Department of Orthodontics and Pediatric Dentistry, School of Dentistry, The University of Michigan, Ann Arbor, MI.; 2 Research Associate, Department of Surgery and Translational Medicine, The University of Florence, Florence, Italy; and Thomas M. Graber Visiting Scholar, Department of Orthodontics and Pediatric Dentistry, School of Dentistry, The University of Michigan, Ann Arbor, MI.; 3 Assistant Professor, Department of Orthodontics and Pediatric Dentistry, School of Dentistry The University of Michigan, Ann Arbor, MI.; 4 Instructor Professor, Guarulhos University, Guarulhos, Brazil.; 5 Thomas M. and Doris Graber Endowed Professor Emeritus (Active), Department of Orthodontics and Pediatric Dentistry, School of Dentistry. Professor Emeritus of Cell and Development Biology, School of Medicine; and Research Professor Emeritus, Center of Human Growth and Development, The University of Michigan, Ann Arbor, MI.

**Keywords:** Suture, Tomography, Orthopedics, Rapid maxillary expansion

## Abstract

Rapid maxillary expansion (RME) primarily involves the mechanical opening of the midpalatal suture of the maxillary and palatine bones. The fusion of the midpalatal suture determines the failure of RME, a common event in late adolescents and young adults. Recently, the assessment of the maturation of midpalatal suture as viewed using cone beam computed tomography (CBCT) has been introduced. Five maturational stages of the midpalatal suture have been presented: Stage A = straight high-density sutural line, with no or little interdigitation; Stage B = scalloped appearance of the high-density sutural line; Stage C = two parallel, scalloped, high-density lines that lie close to each other, separated in some areas by small low-density spaces; Stage D = fusion of the palatine bone where no evidence of a suture is present; and Stage E = complete fusion that extends also anteriorly in the maxilla. At Stage C, less skeletal response would be expected than at Stages A and B, as there are many bony bridges along the suture. For patients at Stages D and E, surgically assisted RME would be necessary, as the fusion of the midpalatal suture already has occurred either partially or totally. This diagnostic method can be used to estimate the prognosis of the RME, mainly for late adolescents and young adults for whom this procedure is unpredictable clinically.

## RESUMO

A expansão rápida da maxila (ERM) essencialmente consiste na abertura mecânica da sutura palatina mediana tanto nos ossos maxilares quanto nos ossos palatinos. A fusão da sutura palatina mediana determina o insucesso da ERM, um evento comum na adolescência tardia e fase adulta jovem. Recentemente, propôs-se a avaliação da maturação da sutura palatina mediana em tomografias computadorizadas de feixe cônico (TCFC), sendo apresentados cinco estágios maturacionais: Estágio A = linha sutural de alta densidade retilínea, sem ou com suave interdigitação; Estágio B = linha sutural de alta densidade, com aspecto tortuoso; Estágio C = duas linhas de alta densidade, paralelas e curvilíneas, que se aproximam em algumas regiões e, em outras, são separadas por espaços de baixa densidade; Estágio D = a fusão ocorreu no osso palatino, onde não há evidência de sutura; e Estágio E = fusão completa, que se estende anteriormente na maxila. No Estágio C, esperam-se menores efeitos esqueléticos da ERM, comparado aos Estágios A e B, visto que há muitas pontes ósseas ao longo da sutura. Para pacientes nos Estágios D e E, a expansão rápida da maxila assistida cirurgicamente (ERMAC) seria necessária, já que a fusão da sutura palatina mediana ocorreu parcial ou totalmente. Esse método de diagnóstico pode ser utilizado para estimar o prognóstico da ERM, principalmente na adolescência tardia e fase adulta jovem, período em que esse procedimento ainda apresenta-se imprevisível clinicamente.

## INTRODUCTION

Rapid maxillary expansion (RME) is a routine procedure performed in orthodontic practice that is used to widen maxilla in order to correct posterior crossbite and maxillary crowding.^1^ ^-^ ^4^ For Class III malocclusion, the RME has been employed in combination with an orthopedic facial mask to produce both skeletal and dentoalveolar effects.^5^

**Figure 1:**
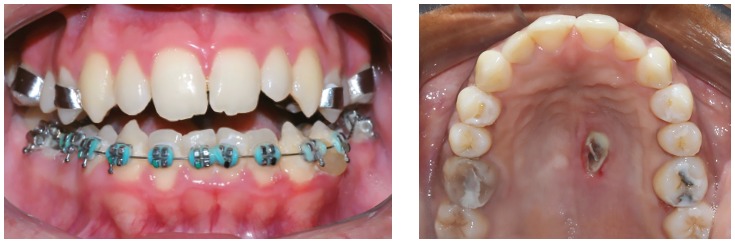
Side-effects after the RME failure: accentuated buccal inclination of the maxillary posterior teeth and necrosis of the palate.

In 1860, Angell^1^ introduced the concept that the maxilla could be expanded by means of opening the midpalatal suture. However, only after the landmark study conducted by Haas,^3^ ^,^ ^4^ 100 years later, this therapy became routine in Orthodontics. Clinically, RME has been indicated for growing patients, as the failure of this therapy is relatively common in adults because of the fusion of the sutures. Serious pain, mucosal ulceration or necrosis, and accentuated buccal tipping and gingival recession around the posterior teeth^6^ ^-^ ^10^ have been observed after RME failure (Fig 1).

The typical clinical criterion for making the choice between conventional RME and surgically assisted rapid maxillary expansion (SARME) is the chronological age of the patient. However, there is no consensus in the literature about the age for indication of SARME. SARME has been recommended for patients older than 14,^11^ 16,^12^ 20,^13^ or 25 years of age.^14^ A difference between genders was reported in one study^15^ in which SARME was indicated for females older than 20 years and in males older than 25 years.

In the same way, the start and gradual fusion of the midpalatal suture presents great variability according to the age and gender of the patient. During the maturation process, the interdigitation of the midpalatal suture increases,^16^ ^,^ ^17^ and the fusion begins in the posterior area, progressing from palatine bone anteriorly to the maxilla.^16^ ^,^ ^18^ Persson and Thilander^18^ have verified fusion of the midpalatal suture in the posterior palate of a 15-year-old female and a 21-year-old male. On the other hand, no fusion of the midpalatal suture has been observed in patients of ages 27 and 32 years,^18^ 54 years,^16^ and even 71 years.^19^

These histological results do not match with clinical experience, however, which is known to be very difficult to obtain success using conventional RME in individuals older than 25 years of age.^20^ Some authors, still, have demonstrated success using conventional RME in adults.^15^ ^,^ ^21^ ^-^ ^23^ Hence, the fusion of the midpalatal suture apparently is not related directly to chronological age, particularly in late adolescents and young adults.^16^ ^,^ ^18^ ^-^ ^20^ ^,^ ^24^ For these patients, an individualized clinical assessment of the maturation of the midpalatal suture is recommended before RME, in order to make the choice between conventional RME or SARME. 

The individual assessment of the midpalatal suture prior to RME on occlusal radiographs has been introduced by Revelo and Fishman.^25^ Nevertheless, some years later, Wehrbein and Yildizhan^20^ demonstrated histologically that occlusal radiographs are unreliable for the diagnosis of the fusion of the midpalatal suture because of the superimposition of the vomer and the structures of the external nose in the midpalatal area. 

Because of the absence of clinical parameters for predicting RME success in late adolescents and young adults, Angelieri et al^26^ have introduced an individual assessment of the maturation of the midpalatal suture using cone-beam computed tomography (CBCT) images of the suture. The present article presents this classification method for the assessment of the midpalatal suture in an individual patient, besides discussing the clinical implications for using this approach prior to initiating RME in older adolescent and adult patients.

### Classification of midpalatal suture maturation on CBCTs

For the evaluation of midpalatal suture maturation on CBCTs, several types of commercially-available softwares may be used, allowing visualization of the images in axial, sagittal, and coronal views. Also it is important that this software facilitate the easy adjustment of the head orientation of the patient in the CBCT image. Usually, the software used by our group in these types of investigations is Invivo5 (Anatomage, San Jose, CA, USA). 

**Figure 2:**
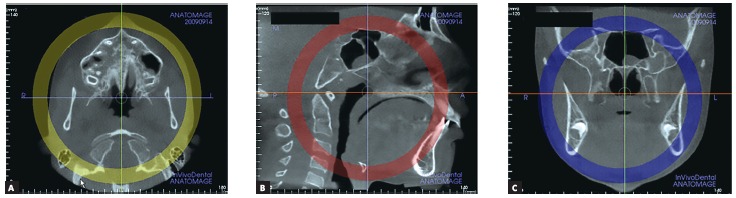
Orientation of head position in the axial (A), sagittal (B) and coronal planes (C). Source: Angelieri et al,^26^ 2013.

Firstly, the head orientation should be oriented in natural head position in all three planes of space. The cursor (the position indicator) of the image analysis software is positioned at the midsagittal plane of the patient in both coronal and axial views (Fig 2). In the sagittal view, the patient’s head is adjusted so that the anteroposterior long axis of the palate is horizontal. The vertical and horizontal cursors should be positioned in the center of palate in axial, coronal, and sagittal views.

**Figure 3:**
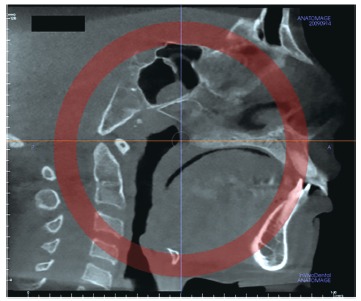
Selection of the most central cross-sectional slice in the superior-inferior dimension, to assess the midpalatal suture maturation.

The most central axial cross-sectional slice is used for assessment of the midpalatal suture maturation. For selecting this slice in the sagittal plane (on the mid-sagittal cross-sectional slice), the palate should be positioned horizontally, parallel to the software’s horizontal orange line. After placing this horizontal line along the palate, the most central cross-sectional slice in the superior-inferior dimension (i.e., from the nasal to the oral surface) is utilized for classification of the maturational stage of the midpalatal suture (Fig 3). 

**Figure 4:**
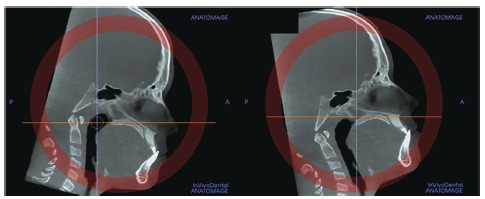
In palates that are curved, two central cross-sectional axial images should be examined. Source: Angelieri et al,^26^ 2013.

**Figure 5:**
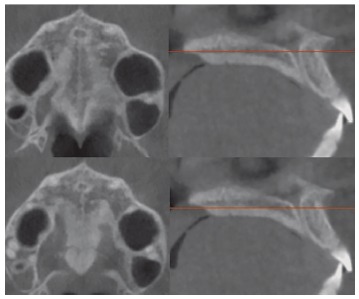
In thick palates, the two most central axial slices should be evaluated and the more mature cross-sectional slice should be evaluated. Source: Angelieri et al,^26^ 2013.

However, some individuals present a curved palatal contour, and for them, the palate should be analyzed in two separate central cross-sectional axial slices, one from posterior and another from anterior region of the midpalatal suture, separately (Fig 4). Furthermore, for subjects who presented with a thicker palate, the palate should be evaluated in the two most central axial slices (Fig 5). The more matured central cross-sectional axial slice should be considered.

Based on the histological findings of the morphology of the midpalatal suture observed during growth,^17^ ^,^ ^24^ ^,^ ^27^ ^-^ ^29^ five maturational stages were identified, as follows: 

### Stage A

**Figure 6:**
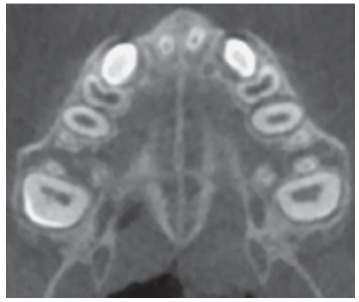
Stage A: the midpalatal suture is almost straight high-density line.

In this stage, the midpalatal suture appears as an almost straight high-density sutural line with no or little interdigitation(Fig 6).^17^ ^,^ ^19^ ^,^ ^28^ ^,^ ^30^

### Stage B

**Figure 7:**
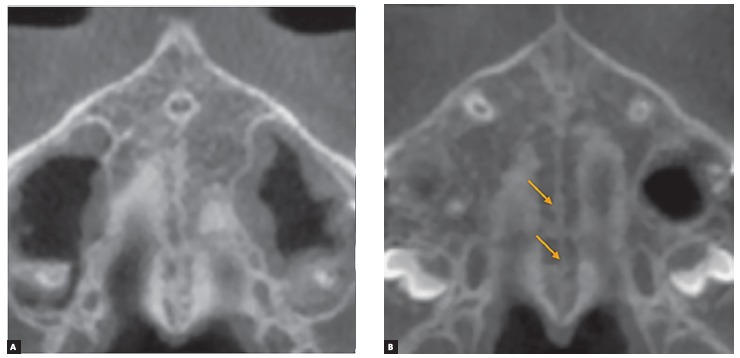
Stage B appears as a scalloped high-density line (A); or in some areas, two parallel, scalloped high-density lines close to each other and separated by small low-density spaces - arrows in B.

At stage B, the midpalatal suture becomes irregular, as one scalloped high-density line (Fig 7A). Usually, in this stage there are some small areas where two parallel, scalloped, high-density lines lie close to each other and are separated by small low-density spaces (Fig. 7B).^19^ ^,^ ^30^

### Stage C

**Figure 8:**
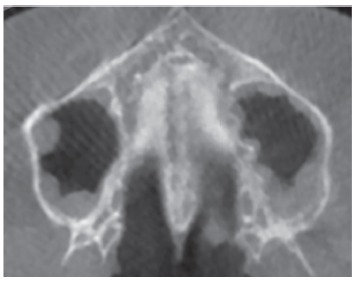
Stage C is characterized as two parallel, scalloped high-density lines close to each other and separated by small low-density spaces in either a straight or an irregular pattern.

At Stage C, the midpalatal suture can be visualized as two parallel, scalloped, high-density lines that are close to each other, separated by small low-density spaces in the maxillary and palatine bones (between the incisive foramen and the palatomaxillary suture and posterior to the palatomaxillary suture). The suture can present either a straight or irregular pattern (Fig 8).

### Stage D

**Figure 9:**
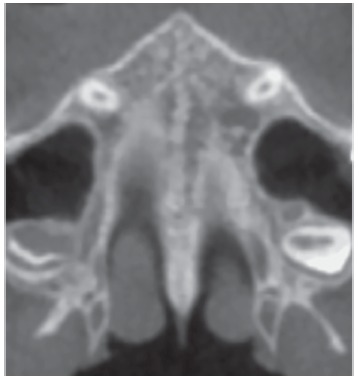
Stage D: in the palatine bone, the midpalatal suture cannot be visualized and the parasutural bone density is increased.

In this stage, the fusion of the midpalatal suture has occurred in the palatine bone, so the midpalatal suture cannot be visualized in the palatine bone, as usually the fusion happens from posterior to anterior portion^16^ ^,^ ^18^ (Fig 9). It is important to stress that the parasutural bone density is increased (high-density bone) compared to the density of the maxillary parasutural bone. In the maxillary portion, the midpalatal suture still appears as two high-density lines separated by small low-density spaces. 

### Stage E

**Figure 10:**
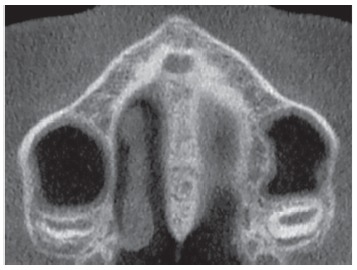
At Stage E, the midpalatal suture is not visible in at least a portion of maxilla.

At stage E, the midpalatal suture cannot be visualized in at least a portion of the maxilla,^28^ ^,^ ^29^ once at least one partial fusion of this suture has happened in the maxilla (Fig 10). The parasutural bone density is increased, with the same level as in other regions of the palate.^19^

**Figure 11:**
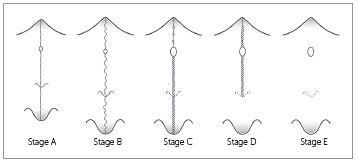
Schematic drawing (by Chris Jung) of the maturational stages of the midpalatal suture. Source: Angelieri et al,^26^ 2013.

All maturational stages of the midpalatal suture are represented in the schematic drawing depicted in Figure 11.

### Clinical implications of midpalatal sutural maturation on CBCTs

**Figure 12:**
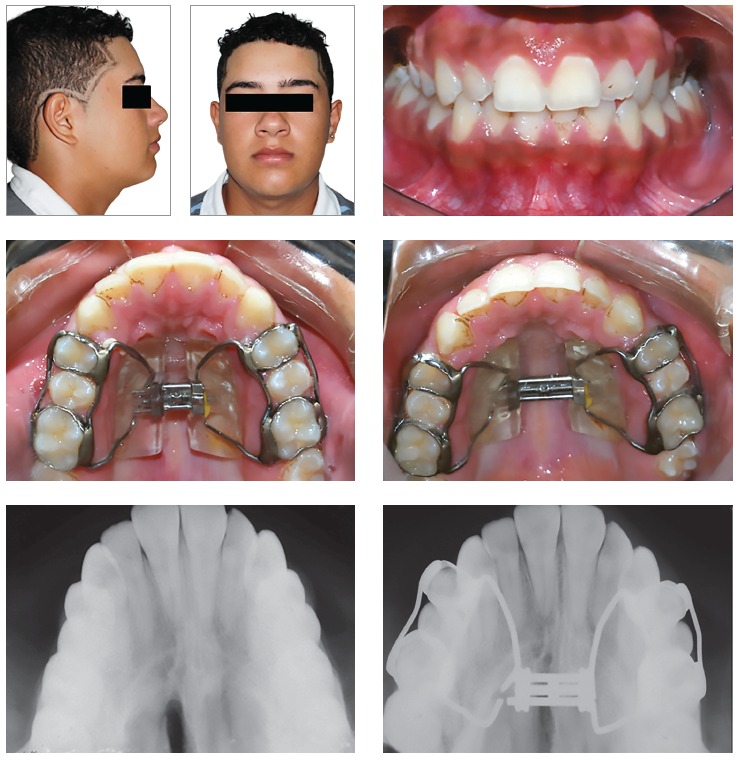
16-year-old boy treated with Haas expander. There was the failure of RME. Source: Angelieri et al,^34^ 2015.

**Figure 13:**
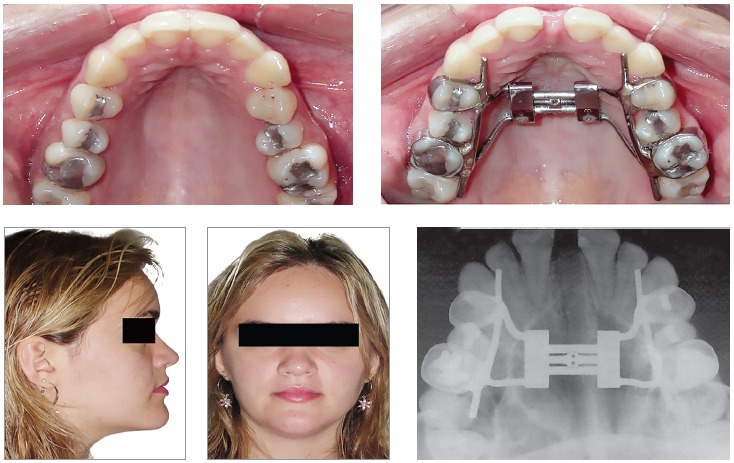
Successful RME in a 16-year-old girl treated with Hyrax-expander.

The presence of posterior crossbite or atresia of the maxilla in late adolescents or young adults has been a challenge for orthodontists. The clinical choice between conventional RME or SARME implies possible unnecessary surgical procedures - demanding costs and risks for patients - or side-effects of conventional RME failure as severe pain, mucosal ulceration or necrosis, accentuated buccal tipping and gingival recession in the posterior teeth.^6^ ^-^ ^10^ There are no clinical parameters for this difficult decision; histological and micro-CT studies have demonstrated that chronological age and gender are not a reliable parameter for the fusion of the midpalatal suture^16^ ^,^ ^18^ ^,^ ^19^ ^,^ ^20^ ^,^ ^24^ (Figs 12 and 13).

CBCT imaging facilitates three dimensional visualization of the oral and maxillofacial structures, allowing the evaluation of the midpalatal suture maturation^26^ without the overlay of the vomer and other external structures of nose on the midpalatal region, as occurs on occlusal radiographs.^20^

It is interesting that the five maturational stages identified on CBCTs corroborate with the histological findings of midpalatal suture maturation. In a landmark study, Melsen^17^ observed that in the juvenile period (usually up to 10 years of age), the midpalatal suture is broad and Y-shaped in frontal sections.^28^ ^,^ ^29^ From 10 to 13 years of age, this suture appears with a squamous path, becoming wavier with increased interdigitation at ages 13 to 14 years. These descriptions match Stages A and B, respectively, with the increase of the interdigitation characterizing the more matured stage. 

The fusion of the midpalatal suture has been described in several histological studies. The fusion process of the midpalatal suture begins with bone spicules from suture margins along with “islands” (i.e., masses of acellular tissue and inconsistently-calcified tissue) in the middle of the sutural gap.^18^ ^,^ ^19^ ^,^ ^24^ ^,^ ^28^ These spicules are present in many places along the suture, and they increase with maturation.^18^ ^,^ ^27^ The spicules appear as many scalloped areas that are close to each other and yet are separated in some zones by connective tissue.^16^ ^,^ ^20^ This description is compatible with Stage C, in which many bony bridges can be visualized along the suture, leading to more resistance for conventional RME. Probably, RME performed in patients at Stages A and B would have less resistance forces and more skeletal effects than when performed during Stage C. 

Angelieri et al.^31^ demonstrated that the midpalatal suture maturation is related to skeletal growth, since a high correlation coefficient was observed between the cervical vertebra maturation and maturational stages of the midpalatal suture. According to the results, the prepubertal stages (cervical stages CS1 and CS2) are reliable indicators for stages A and B of midpalatal suture maturation. In the pubertal stage (CS3), probably the patient will present the midpalatal suture at stage C. Considering the presence of many bony bridges along the midpalatal suture at stage C, these findings corroborate the results of Baccetti et al,^32^ who observed more favorable skeletal changes from RME in prepubertal patients compared to postpubertal patients. 

Furthermore, Krukemeyer^33^ evaluated the correlation among response to RME, maturational stages of the midpalatal suture, and the stage of cervical vertebral maturation (CVM). The maturational stages of the midpalatal suture and CVM stages were correlated inversely with sutural expansion, i.e. the less mature the patient, the greater was sutural expansion, with more skeletal than dentoalveolar effects of RME. 

**Figure 14:**
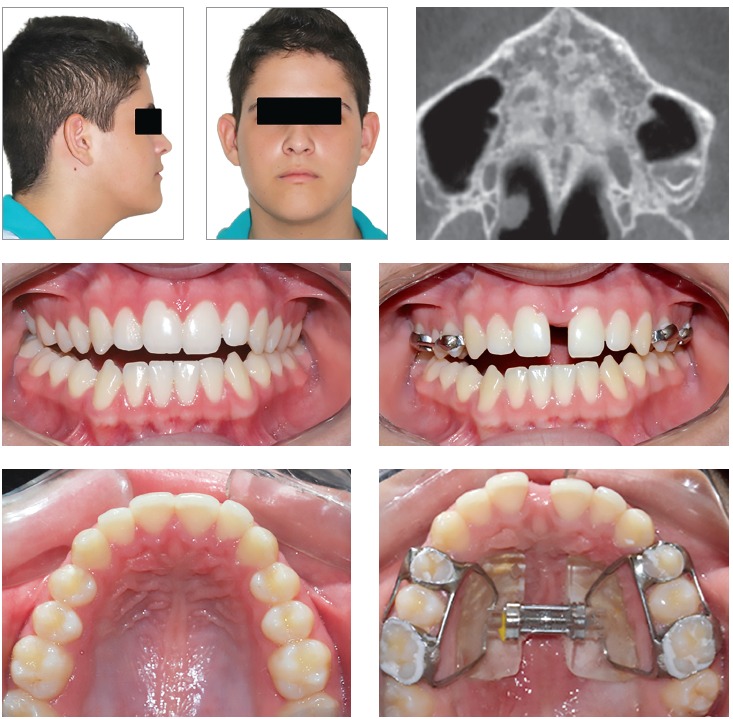
A 15-year-old boy patient at Stage C. Conventional RME still was possible. Source: Angelieri et al,^34^ 2015.

On the other hand, it is important to stress that, in spite of increased sutural resistance to conventional RME at Stage C, the widening of maxilla orthopedically with no surgical interventional still is possible (Fig. 14). This procedure should be initiated immediately, due to the start of fusion of the palatine portion of the midpalatal suture might being imminent.^34^

With the maturation of midpalatal suture, there is an increase in interdigitation.^16^ ^,^ ^17^ As mentioned previously, sutural fusion happens earlier in the posterior region and subsequently progresses toward the anterior,^16^ ^,^ ^18^ with resorption of cortical bone in the sutural ends and the subsequent formation of cancellous bone.^28^ ^,^ ^29^ When patients are at Stage D, it is possible to visualize the interincisal diastema promoted by RME, even though no widening of the palate will have occurred posteriorly. The fusion of the palatine (Stage D) or/and maxillary portions (Stage E) of the midpalatal suture hampers the expansive forces of conventional RME; these patients are treated more effectively by surgically-assisted RME.^34^

Angelieri et al^26^ evaluated a sample of 140 subjects from 5.6 to 58.4 years of age and verified fusion of the midpalatal suture on CBCTs in girls older than 11 years (Stages D and E) and boys older than 14 years (Stage D). Clinically, this sexual dimorphism in the fusion of the midpalatal suture has been noted, with females usually maturing earlier than males. Furthermore, the majority of the adults presented fusion of the midpalatal suture in the palatine or/and maxillary portions. 

Interestingly, these results corroborate the clinical findings of RME failure in late adolescents, mainly in females and adults. However, no fusion of the midpalatal suture has been verified histologically in some adults in their third through seventh decades of life.^16^ ^,^ ^18^ ^,^ ^19^ In these histological studies, only the frontal sections of anterior portions of the midpalatal sutures were evaluated. Considering that the maturation of the midpalatal suture occurs progressively from the posterior to the anterior regions, those subjects could have presented patent midpalatal sutures in the anterior portion and possibly fusion of the posterior portion of the midpalatal suture. 

Only Persson and Thilander^18^ have analyzed histologically the palatine portion of the midpalatal suture, verifying fusion of this suture in subjects ranging from 15 to 19 years of age. Thus it is essential that the evaluation of the midpalatal suture is anteroposterior, along its long axis, with no overlay of adjacent structures, to diagnose properly the stage of maturation of the midpalatal suture.

Still, Angelieri et al.^31^ have verified that for patients at postpubertal stages of cervical vertebral maturation (CS4), the midpalatal sutural stage is unpredictable. Thus for these patients, an assessment of the midpalatal suture on CBCT is recommended before the clinical decision between conventional RME or surgically assisted RME is made. In an early study, Angelieri et al.^26^ observed no fusion of the midpalatal suture in some adults (Stages B or C), a finding that probably would lead to treatment with conventional RME. The success of the conventional RME in some adults has been shown by some studies.^15^ ^,^ ^21^ ^-^ ^23^ However, other factors should be evaluated for successful conventional RME in adults, such as fusion of other circummaxillary sutures. 

Therefore, this individual assessment of midpalatal suture maturation has the potential to allow the development of a reliable diagnostic guidance for the prediction of RME success or failure, mainly for late adolescent and young adult patients for whom the prognosis of RME is questionable. Future studies would be encouraged to analyze the clinical meaning of the different maturational stages of the midpalatal suture and the application of this method to other circummaxillary sutures. 

## CONCLUSIONS

Making the choice between conventional or assisted surgically RME in late adolescents and young adults historically has been a difficult decision for clinicians. The individual assessment of midpalatal suture maturation on CBCT images may be a promising predictor for conventional or assisted surgically RME, avoiding unnecessary surgery, accentuated dental tipping, gingival recession, severe pain, and even necrosis of the palate. 
